# N-Hexane neuropathy: from addiction to disability!

**DOI:** 10.1192/j.eurpsy.2023.1385

**Published:** 2023-07-19

**Authors:** L. Hlioui, R. Zouari, D. Ben Mohamed, M. Z. Saied, J. Ketata, F. Nabli, S. Ben Sassi

**Affiliations:** neurology department, national institute of neurology of Tunis, Tunis, Tunisia

## Abstract

**Introduction:**

Voluntary poisoning with neurotoxic products in order to achieve euphoria is common especially among young people. Neurological complications are quite likely and can be serious and irreversible.

**Objectives:**

We aim to describe the peripheral neuropathies secondary to N-Hexane intoxication in a Tunisian population.

**Methods:**

A retrospective descriptive study was carried out in our department of neurology in the NationalInstitute of Neurology of Tunis including patients diagnosed with N-Hexane neuropathy. All patientshad a history of a N-Hexane exposure. The diagnosis was confirmed after excluding other etiologiesthrough appropriate investigations. Clinical and para-clinical data as well as follow-up were assessed.

**Results:**

We selected 38 patients with a mean age of 22.7 years [14-36]. Among them, 37 were glue-sniffer and 1 had a voluntary toxic exposure to paint. An associated cannabis consumption was found in 6 patients. All of them had a low socio-economic background and 17 were unemployed. Time to onset of neurological signs ranged from 5 months to 11 years. The clinical exam showed a quadriparesis (15,7%), a paraparesis (58%), sensory involvement (55,2%) amyotrophy (40%) and abolished tendon reflexes in lower limbs (81,5%). Swallowing disorder and optic neuritis were found in one case. The electroneuromyogram revealed an axono-demyelinating sensory-motor polyneuropathy (PN) in 16 cases and a demyelinating motor PN in 9 cases. Vitamin therapy, motor rehabilitation and psychotherapy sessions have been indicated. Only 6 patients showed slight clinical improvement after withdrawal. The rest of our patients did not quit; 84% of them became bedridden.

**Conclusions:**

Glue-sniffer related neuropathy is very common in our country especially in adolescents and young adults with low socio-economic background. The neurological outcome is serious and usually irreversible if exposure is persistent.

**Disclosure of Interest:**

None Declared

